# Combination of percutaneous endoscopic lumbar discectomy and platelet-rich plasma hydrogel injection for the treatment of lumbar disc herniation

**DOI:** 10.1186/s13018-023-04093-w

**Published:** 2023-08-21

**Authors:** Lidong Zhang, Chengliang Zhang, Dajiang Song, Gang Chen, Lei Liu

**Affiliations:** Department of Orthopedics, The Affiliated Shuyang Hospital of Xuzhou Medical University, 9 Yingbin Road, Suqian, 223600 China

**Keywords:** Lumbar disc herniation, Platelet-rich plasma, Percutaneous endoscopic lumbar discectomy

## Abstract

**Objective:**

To determine the safety and efficacy of percutaneous endoscopic lumbar discectomy (PELD) combined with platelet-rich plasma (PRP) hydrogel injection in patients with lumbar disc herniation (LDH).

**Methods:**

A total of 98 consecutive patients with LDH who underwent either PELD combined with PRP hydrogel injection or PELD alone were reviewed. This retrospective study was performed between January 2019 and January 2021. Clinical outcomes were compared in the visual analog scale (VAS) for low back pain and leg pain, Oswestry disability index (ODI), Japanese Orthopaedic Association (JOA) scores, and Macnab criteria. Intervertebral disc height on MRI was measured, and the Pfirrmann grade classification was used pre-operatively and post-operatively.

**Results:**

No severe adverse events were reported during an 18-month follow-up period. VAS scores for back pain were decreased at 1 month, 3 months, and 18 months in the treatment group than that in the control group. JOA score and ODI in the treatment group at 3-month and 18-month follow-up was lower than that in the control group (*P* < 0.05). The excellent and good rate of the Macnab criteria was 92.0% (46/50) in the treatment group and 89.6% (43/48) in the control group (*P* > 0.05). The comparison of Pfirrmann grading and disc height at 18-month follow-up showed significant difference in two groups (*P* < 0*.*05). The recurrence of LDH in the treatment group was lower than that in the control group (*P* < 0*.*05).

**Conclusions:**

We suggest that PELD combined with PRP hydrogel injection to treat patients with LDH is a safe and promising method. PRP injection was beneficial for disc remodelling after PELD.

## Introduction

Lumbar disc herniation (LDH) presents a trend of a low age and high morbidity and becomes a common and frequent disease that seriously affects people's daily life [1, 2]. Most patients with LDH recover in 1 to 3 months with conservative treatment. However, about 20% of patients have recurrent LDH [3]. Traditional open surgery serves as an effective method, but is along with several disadvantages, including post-operative back pain and a long-period recovery [4, 5]. Consequently, the minimally invasive technique draws increasing focus from surgeons and patients around the world. Percutaneous endoscopic lumbar discectomy (PELD) has been perceived to solve lumbar discectomy with the advantages such as soft tissue protection, less blood loss, and shorter hospital stays [2, 6, 7].

LDH is characterized by degeneration of the intervertebral disc. During the process of disc degeneration, the fissures of the annulus fibrosus (AF) cause the migration of the nucleus pulposus. It leads to the release of pro-inflammatory cytokines which initiate chemical sensitization of the nociceptors found in the outer AF [8, 9]. In recent years, autologous cell therapies, including platelet-rich plasma (PRP) and bone marrow concentrate, have been studied extensively in the treatment of degenerative lumbar disease [10–12]. PRP is found to be rich in growth factors that promote tissue repair and reconstruction. Studies have also revealed that these growth factors are agents in facilitating cell migration, proliferation, and synthesis of extracellular matrix proteins and collagen [13, 14]. Additionally, studies have demonstrated that concentrated PRP has a positive recovery effect on degenerative discs [15].

Percutaneous injection of PRP in the treatment of low back pain in vitro has yielded promising results. But there is little literature on the safety and effectiveness of PELD combined with PRP for patients with LDH. PELD can remove the prolapsed nucleus pulposus (NP) and protruding AF, However, the burning of the electrocoagulation during the operation will also damage the NP and AF, which will lead to accelerated disc degeneration in the patient, and even cause the recurrence of herniation. In the present study, we evaluated the safety and effectiveness of PRP hydrogel injection for patients who underwent PELD to determine whether it could offer a better therapeutic effect.

## Materials and methods

### Ethics and patient selection

This retrospective study was approved by the Institutional Review Board of Xuzhou Medical University. Patients were informed of the possible risks of the two methods and provided written informed consent. This study was conducted following the principles of the Declaration of Helsinki.

Patients with LDH who underwent PELD with or without PRP hydrogel injection between January 2019 and January 2021 were assessed for eligibility. The type of procedure was chosen according to each patient. Ninety-eight consecutive patients were included in our study. Of these patients, 50 underwent PELD combined with PRP hydrogel injection and 48 underwent PELD alone. All patients were followed up for 18 months. The inclusion criteria were: (1) symptoms and signs of leg pain and/or low back pain, matched with imaging results; (2) failure of conservative treatments after 6 weeks; (3) age under 60 years old; and (4) platelet count > 120 × 10^9^/L. The exclusion criteria were: (1) more than one segment LDH; (2) calcification was found in the segment; (3) cauda equina syndrome was found; (4) LDH with instability, infection, tumour, or deformity; and (5) previous lumbar surgery history.

### PRP preparation

A PRP preparation kit (Wego Co. Ltd., Shandong, China) was used for injection. A volume of 30 ml of blood was taken from the vein before the surgery. Then, the sample was centrifuged to stratify the layer containing leukocytes and platelets. Subsequently, excess erythrocytes were removed. Finally, second centrifugation was performed to obtain the PRP hydrogel after the supernatant was removed. The volume of PRP hydrogel for the injection was 3.5–4 ml.

### Operative procedure

**Fig. 1:**
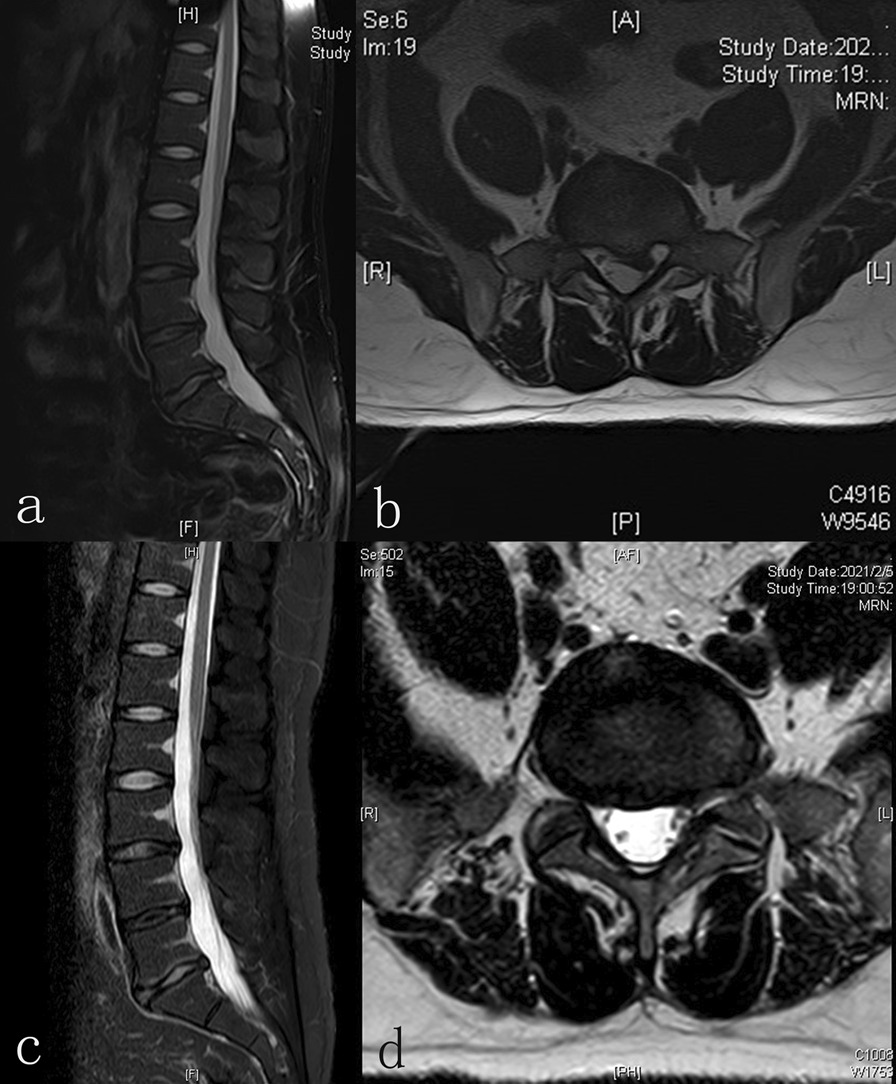
Representative magnetic resonance imaging (MRI). A patient was diagnosed with L5-S1 lumbar disc herniation (LDH). A sagittal and cross-sectional MRI revealed L5-S1 LDH pre-treatment (**a**, **b**). MRI sagittal and cross-sectional image of improvement after percutaneous endoscopic lumbar discectomy (PELD) combined with platelet-rich plasma (PRP) hydrogel injection at L5-S1 at 1 year post-treatment (**c**, **d**)

**Fig. 2:**
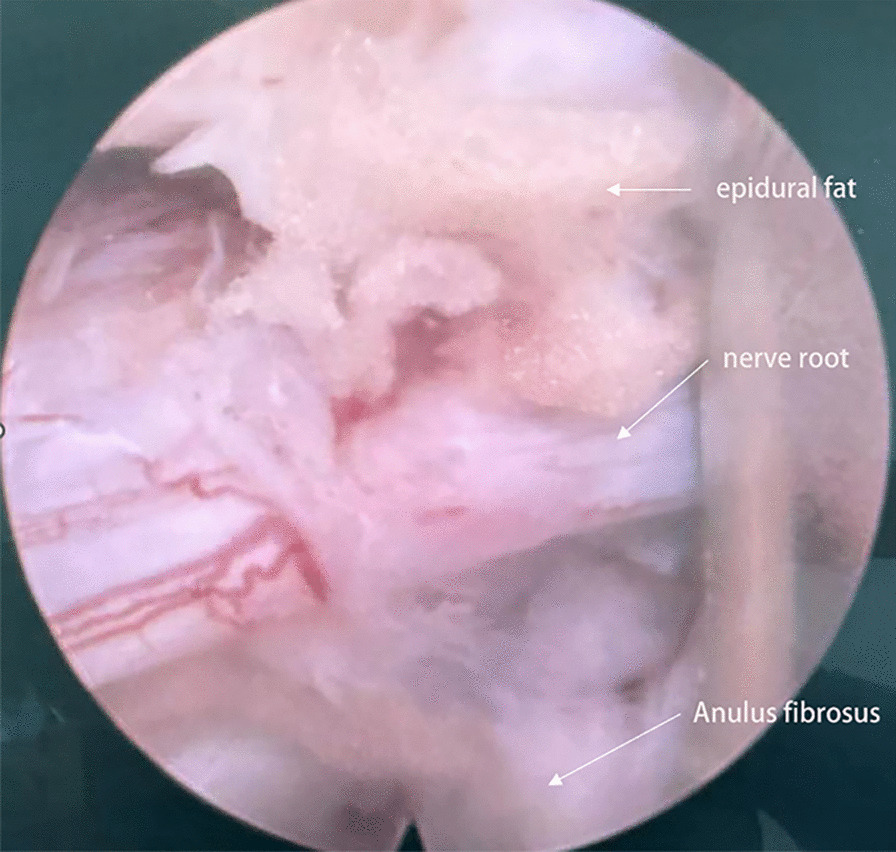
Endoscopic shot after transforaminal lumbar endoscopic discectomy

Patients were placed in a lateral position. The responsible segment was confirmed by C-arm fluoroscopy. The puncture site was set at 5 ~ 7 cm next to the posterior midline after local anaesthesia. An 18-G puncture needle was inserted from the marked puncture site to the lateral aspect of the superior articular process under C-arm monitoring. The puncture needle was removed after inserting the guidewire. An approximately 0.7 cm cut was made, and a series of expanding dilators were inserted sequentially. Finally, a cannula was inserted which by a trephine was prepared to remove the part of the superior articular process. The position of the cannula was confirmed under C-arm monitoring. The cannula was irrigated with saline. After removal of the herniated NP, an annuloplasty was performed by a bipolar radiofrequency ablator. The nerve root was explored to be relieved (Figs. 1, 2). The PRP hydrogel was injected into the site where annuloplasty was done under endoscopic monitoring after drawing out the saline. PELD was performed without PRP injection in the control group. The cannula was removed, and the incision was sutured.

### Outcome assessment

Demographic data, duration of pain, and patient-reported outcomes were collected. Visual analog scale (VAS) [16] for low back pain and leg pain was recorded at baseline and at 1 week, 3 months, 6 months, and 18 months after surgery for clinical assessment. Japanese Orthopaedic Association (JOA) scores [17] and Oswestry disability index (ODI) [18] were documented to evaluate patients’ lumbar function at baseline and at 3 months, 6 months, and 18 months after surgery. The Macnab criteria [19] were used to grade the patient satisfaction as excellent, good, fair, or poor.

Intervertebral disc height of the anterior, midpoint, and posterior margin of the operative disc were measured on MRI at baseline and 18 months after surgery. The average intervertebral disc height was calculated. The Pfirrmann grade classification [20] was used to evaluate the degeneration of the intervertebral disc.

### Statistical analysis

Frequencies and percentages are presented for discrete variables, while continuous variables are reported as means and standard deviations. A t-test was used for continuous variables, and the Chi-squared test was used for discrete variables. Statistical significance was defined as *P* < 0.05. SPSS 23.0 software (IBM Corp., NY, USA) was used for data analysis.

## Results

Ninety-eight patients were reviewed between January 2019 and January 2021 comprising 54 males and 44 females. The mean age was 41.5 ± 9.8 years in the treatment group and 45.8 ± 11.0 years in the control group. The duration of disease was 12.1 ± 10.1 months in the treatment group and 15.3 ± 8.5 months in the control group. The platelet concentration in the treatment group was 212.9 ± 48.1, and in the control group, it was 228.3 ± 52.3. BMI was 21.3 ± 3.1 in the treatment group and 23.4 ± 2.9 in the control group. No significant differences in baseline characteristics were noted between the two groups (Table 1). The affected levels included L3/4, L4/5, and L5/S1. No significant difference in baseline characteristics was found between the two groups (Table 1).

**Table 1:** Baseline characteristics in two groups (mean ± SD)

	The treatment group, *n* = 50	The control group, * n* = 48	*P* value
Age (year)	41.5 ± 9.8	45.8 ± 11.0	0. 262
Gender (–)			0.8569
Male	28 (56%)	26 (54.2%)	
Female	22 (44%)	22 (45.8%)	
Duration of disease (month)	12.1 ± 10.1	15.3 ± 8.5	0.381
Levels			0.925
L3/4	9 (18%)	8 (16.7%)	
L4/5	18 (36%)	16 (33.3%)	
L5/S1	23 (46%)	24 (50.0%)	
Platelet (× 10^9^/L)	212.9 ± 48.1	228.3 ± 52.3	0.298
BMI (kg/m^2^)	21.3 ± 3.1	23.4 ± 2.9	0.392

Table 2 shows the outcomes of lumbar function in two groups. A comparison of JOA, ODI, and VAS scores before surgery revealed no statistical difference (*P* > 0*.*05). No difference was found in VAS scores for leg pain at 1 week, 1 month, 3 months, and 18 months in two groups. VAS scores for back pain were decreased at 1 month, 3 months, and 18 months in the treatment group than that in the control group. JOA score and ODI in the treatment group at 3-month and 18-month follow-up were lower than that in the control group (*P* < 0*.*05). The excellent and good rate of the Macnab criteria was 92.0% (46/50) in the treatment group and 89.6% (43/48) in the control group, which showed no significant difference (*P* > 0*.*05).

**Table 2:** VAS, ODI, and JOA scores before and after surgery in two groups (mean ± SD)

	The treatment group	The control group	*P* value
*VAS for leg pain*			
Pre-operative	7.0 ± 1.9	7.3 ± 2.1	0.581
Post-operative			
1 week	1.6 ± 1.5	1.7 ± 1.6	0.692
1 month	1.5 ± 0.9	1.3 ± 1.1	0.466
3 months	1.2 ± 0.8	1.2 ± 0.9	0.710
18 months	1.1 ± 0.9	1.0 ± 1.0	0.714
*VAS for low back pain*			
Pre-operative	5.1 ± 2.0	4.8 ± 2.5	0.438
Post-operative			
1 week	2.3 ± 1.8	2.4 ± 2.0	0.519
1 month	1.7 ± 1.6	2.3 ± 1.9	0.032*
3 months	1.2 ± 0.9	1.9 ± 1.8	0.014*
18 months	0.9 ± 0.8	1.8 ± 1.7	0.029*
*JOA score*			
Pre-operative	13.3 ± 3.2	12.2 ± 3.4	0.551
Post-operative			
1 month	19.3 ± 2.9	17.9 ± 3.2	0.387
3 months	24.2 ± 2.9	19.3 ± 3.5	0.041*
18 months	26.6 ± 3.0	21.2 ± 3.1	0.030*
*ODI*			
Pre-operative	45.2 ± 21.7	47.3 ± 20.3	0.843
Post-operative			
1 month	17.9 ± 7.2	15.5 ± 6.2	0.681
3 months	15.1 ± 7.9	10.4 ± 5.9	0.038
18 months	7.3 ± 4.2	4.4 ± 3.9	0.017
*The Macnab criteria*			
			0.674
Excellent	28	23	
Good	14	20	
Fair	3	3	
Poor	1	1	

VAS, visual analog scale; ODI, Oswestry disability index; JOA, Japanese Orthopaedic Association; *SD*, standard deviation, *P < 0.05

Mean disc height was 10.50 ± 0.91 mm and 10.25 ± 0.81 mm at baseline and final follow-up in the treatment group, respectively, and 10.41 ± 0.78 mm and 9.32 ± 0.85 mm in the control group with significant difference (*P* < 0*.*05) (Table 3). The comparison of Pfirrmann grading at 18-month follow-up showed significant difference in two groups (*P* < 0*.*05).

**Table 3:** Outcomes of intervertebral disc height and the Pfirrmann grade 18 months after treatment in two groups

	The treatment group, *n* = 50	The control group, *n* = 48	*P* value
Intervertebral disc height			
Before treatment	10.50 ± 0.91	10.41 ± 0.78	0.771
Final follow-up	10.25 ± 0.81	9.32 ± 0.85	0.042
The Pfirrmann grade			
Before treatment			0.169
I	0	0	
II	2	4	
III	31	32	
IV	17	8	
Final follow-up			0.049
I	0	0	
II	1	1	
III	30	17	
IV	19	30	

Two patients in the treatment group showed recurrent radicular pain and underwent revision surgery, and six patients in the control group received revision surgery due to recurrence of LDH. The recurrence rate was 4.0% (2/50) in the treatment group, which is significantly lower than the 12.5% (6/48) in the control group (*P* < 0*.*05). No other severe surgery-related complication occurred.

## Discussion

The purpose of this study was to determine whether the PRP hydrogel injection after the PELD in the treatment of LDH provides more benefit than the PELD alone. Improvement was seen in VAS for back pain, JOA, and ODI scores at 3-month and 18-month follow-up. These results are consistent with the study performed by Yi et al. [21]. No signs of instability, paraesthesia, or muscle weakness were found in all patients.

Common treatments of LDH consist of a combination of methods, such as activity restriction, physical therapy, non-steroidal anti-inflammatory drugs, and analgesic injections, which have shown to alleviate symptoms, but do not change the progression. PRP, as an FDA-approved treatment modality, has been applied successfully for decades by orthopaedic doctors for purpose of musculoskeletal conditions [22]. Favourable findings have been reported in clinical research on the disease of the elbow [23], rotator cuff tendons [24], and knee articular cartilage [25]. The mechanism of PRP's function not only offers a useful matrix for cell multiplication but also provides kinds of bioactive factors such as vascular endothelial growth factor and platelet-derived growth factor which are favourable in recruiting cells such as mesenchymal stromal cells and fibroblasts to the site of damage and stimulating subsequent proliferation and biosynthetic activity [26]. Positive effects of PRP have been demonstrated by in vitro studies of animal and human disc cells [15]. Disc cells demonstrate improved proteoglycan synthesis and AF cell proliferation when cultured with PRP [27]. It appears that PRP shows an inhibitive effect on the detrimental inflammation of TNF-alpha and interleukin-1 on human NP cells [28].

To date, the study on PRP injection combined with PELD surgery is rare. However, there are several articles discussing clinical outcomes following intradiscal injections of PRP in patients with degenerative disc disease. A meta-analysis by Takashi et al. [29] included 5 retrospective studies and concluded that intradiscal PRP injection for degenerative lumbar disc disease results in a statistically significant improvement in VAS with low complication and re-injection rates. The authors of this study agreed that PRP is a targeted annular therapy, so if the endplates have degenerated seriously and the protrusion was significant, PRP hydrogel injection combined with PELD would be of no clinical benefit. Besides, we excluded Grade V annular fissures as the space for PRP hydrogel was inadequate which allowed no odds for possible pro-healing effect. The injected volume of the hydrogel was 3.5-4 ml. When we injected the gel, we detected the outflow the hydrogel through the annuloplasty or the annular fissures. So, the actual hydrogel injected into the disc was usually less than 3.5 ml. But it is hard to determine exactly how many millilitres of hydrogel outflow In this study, the patients who received PRP hydrogel and PELD showed increased improvement in low back pain, JOA, and ODI scores at 3 months post-surgery than those who received PELD alone, suggesting that PRP hydrogel injection may release factors that could prevent inflammation and improve symptoms.

Another purpose of this study was to assess changes in disc degeneration following PRP hydrogel injection by radiographic analyses. Compared to studies on PRP injection for degenerative lumbar disc disease, this research has two differences. The part of the superior articular process was removed and an annuloplasty was performed by a bipolar radiofrequency ablator during the surgery. Together, these two factors may accelerate disc degeneration. A significant restoring effect on disc height was observed, which suggested that PRP hydrogel injection did improve disc remodelling. The Pfirrmann grade may evaluate changes in the structural organization of the intervertebral disc. Significant difference at final follow-up in the Pfirrmann grade was found between two groups, which was consistent with the previous finding [30]. Their study found that intradiscal injection of PRP resulted in improved MRI imaging of the disc. There is a tear in the AF of the patient who underwent PELD, so the injected hydrogel could flow from the tear. Although we made the PRP into a hydrogel state, it is difficult to observe whether the hydrogel comes out of the disc when the surgery is done.

The safety of PRP injection is also the focus of this study. Since PRP is obtained from autologous blood, there is no immune rejection with a low risk of infection and allergic reaction [31]. In addition, PRP has been reported to have antimicrobial properties which can help reduce the risk of infection [32]. No symptoms of nerve root irritation occurred in the PRP group. No drug-related complications or puncture-related injury to the traversing nerve root, exiting nerve root, or dura mater was observed.

There are some limitations to consider for this study. First, it was not a placebo-controlled double-blind study. Second, the optimal injection volume of PRP is debatable to optimize the therapeutic effect. Lastly, 18-month follow-up may not be sufficient to observe clinical differences and the sample size was small. A long-time follow-up and a larger sample size are required for further investigation.

## Conclusions

We suggest that PELD combined with PRP hydrogel injection to treat patients with LDH is a safe and promising method. PRP injection was beneficial for disc remodelling after PELD.

## Data Availability

The data used during the present research are available from the corresponding author on request.
